# NMR Studies of the Interactions between Sialyllactoses and the Polysialytransferase Domain for Polysialylation Inhibition

**DOI:** 10.3390/cimb46060340

**Published:** 2024-06-07

**Authors:** Bo Lu, Si-Ming Liao, Shi-Jie Liang, Jian-Xiu Li, Xue-Hui Liu, Ri-Bo Huang, Guo-Ping Zhou

**Affiliations:** 1 National Engineering Research Center for Non-Food Biorefinery, Guangxi Academy of Sciences, 98 Daling Road, Nanning 530007, China; lubo@gxas.cn (B.L.); simingliao@gxas.cn (S.-M.L.); liangshijie@gxas.cn (S.-J.L.); jianxiuli@gxas.cn (J.-X.L.); 2 Institute of Biophysics, Chinese Academy of Sciences, Beijing 100101, China; xhliu@ibp.ac.cn; 3 Life Science and Technology College, Guangxi University, Nanning 530004, China; 4 Rocky Mount Life Science Institute, Rocky Mount, NC 27804, USA

**Keywords:** cancer cell migration, neuronal cell adhesion molecule (NCAM), polysialic acid (polySia), polysialyltransferase (polyST), polysialyltransferase domain (PSTD), sialylactose (SL), NMR spectroscopy, chemical shift perturbation (CSP)

## Abstract

It is known that sialyllactose (SL) in mammalians is a major source of sialic acid (Sia), which can further form cytidine monophosphate sialic acid (CMP-Sia), and the final product is polysialic acid (polySia) using polysialyltransferases (polySTs) on the neural cell adhesion molecule (NCAM). This process is called NCAM polysialylation. The overexpression of polysialylation is strongly related to cancer cell migration, invasion, and metastasis. In order to inhibit the overexpression of polysialylation, in this study, SL was selected as an inhibitor to test whether polysialylation could be inhibited. Our results suggest that the interactions between the polysialyltransferase domain (PSTD) in polyST and CMP-Siaand the PSTD and polySia could be inhibited when the 3′-sialyllactose (3′-SL) or 6′-sialyllactose (6′-SL) concentration is about 0.5 mM or 6′-SL and 3 mM, respectively. The results also show that SLs (particularly for 3′-SL) are the ideal inhibitors compared with another two inhibitors, low-molecular-weight heparin (LMWH) and cytidine monophosphate (CMP), because 3’-SL can not only be used to inhibit NCAM polysialylation, but is also one of the best supplements for infant formula and the gut health system.

## 1. Introduction

The major components of human milk are proteins, fats, and rich diverse oligosaccharides, including human milk oligosaccharide (HMO), which is indigestible by infants, and they play an important role in promoting the health and growth of infants, as well as defense against infection by various pathogenic agents [1]. The most important kind of oligosaccharides is sialylated milk oligosaccharide (SMO) in HMO, which has multifunctional health benefits.

It is also known that sialyllactose (SL) is the most abundant among SMO [2,3,4]. The biological functions of SL in newborns are known, such as anti-infective activity, immune function, gut maturation, bifidogenic activity [5,6,7], as well as the promotion of intestinal development [8,9]. Previous studies indicate that SL inhibits the activation of vascular endothelial growth factor (VEGF) receptor-2 (VEGFR-2), which is mediated by VEGF. In addition, the growth of endothelial cells, formation of diminished tubes, as well as the arrangement of actin filament of endothelial cells can also be inhibited by SL. 

Moreover, it has also been found that tumor cell angiogenesis with Lewis lung carcinoma, melanoma, and colon carcinoma cells could be prevented by SL. Thus, it has been proposed that SL might be an ideal candidate for the development of anti-angiogenic drugs without any side effects [10].

In mammals, SL is a major source of sialic acid (Sia), a key component of brain gangliosides and an essential nutrient in the development of the brain, synaptic connection, and memory formation [3,11]. Sialic acids or N-acetylneuraminic acids (Neu5Ac) are a kind of keto sugar with a nine-carbon backbone in glycoconjugates [12]. 

The polysialylation of neural cell adhesion molecules (NCAMs) or expression of polysialic acid (polySia) on the NCAMs is performed through the following main steps [13,14,15,16,17,18,19,20]:

Hydrolyzation of SL > Hydrolyzation product: sialic acid (Sia) > Sia is transported to the brain and other issue > Formation of CMP-Sia, an activated precursor of Sia > Synthesis of polySia using CMP-Sia and polysialyltransferases (polySTs).

According to the above steps, it is possible that SL, Sia, CMP-Sia, polySia, and polyST are in one system during the generation process of sialylated glycans, and there may be interactions between them; actually, the interactions between polyST and these ligands are actually interactions between the polysialyltransferase domain (PSTD) and its ligands [21,22,23,24,25,26,27,28,29].

The PSTD is a domain consisting of 32 amino acids, unique in the α2,8-polysialyltransferases (polyST). The PSTD is also called a polybasic domain in polySTs due to containing many basic residues (246K, 248K, 250K, 252R, 259R, 262H, 265R, 272K, 276K, and 277R). The relevant experimental evidence led to the postulation that the electrostatic interaction between the polybasic region and polySia chains may anchor the growing polySia chains to the polySTs during the addition of new sialyl residues to the non-reducing termini of the growing polySia chains [18]. In vitro experiments further showed that the critical residues in the PSTD of ST8Sia IV required for NCAM polysialylation were Lys250 and Arg252 in the N-terminal segment, and Lys272, Ile275, Lys276, and Arg277 in the C-terminal segment of the PSTD, indicating that most basic residues play an important role for NCAM polysialylation [17,18].

Because polySia overexpression on NCAM is related to cancer cell migration, it is necessary to study the inhibition of polySia overexpression. Recent studies based on in vitro and NMR experiments suggested that low-molecular-weight heparin (LMWH) has been found to be an efficient inhibitor due to its stronger binding to the PSTD [26]. 

More recent studies suggested that cytidine monophosphate (CMP) can inhibit polysialylation when CMP-Sia and polySia are mixed, and CMP-Sia may play a role in reducing the gathering extent of polySia chains on the PSTD and may benefit from the inhibition of polysialylation [27]. 

There are two main types of SLs; 3′-SL (3′-N-acetylneuraminyl-D-lactose) and 6′-SL (6′-N-acetylneuraminyl-D-lactose) both have antimicrobial activity. The structures of 3′-SL and 6′-SL consist of the monosaccharide N-acetylneuraminic acid linked to the galactosyl subunit of lactose at the 3 position and 6 position, respectively [30,31].

The contents of 3′-SL are the major SL in infant formula, and 6′-SL is the predominant SL in human breast milk [5,6]. Compared with 3′-SL, the 6′-SL concentration is unstable in human breast milk and is declined after the first month of lactation [9]. This is why 6′-SL is more important than 3′-SL when added to infant formula.

In this study, our interest is to determine whether NCAM polysialylation can be inhibited. If so, what is the binding range of SL on the PSTD? What is the difference in the concentrations of 3′-SL and 6′-SL for the inhibition of NCAM polysialylaton? Which inhibitor is more effective when comparing SLs and another two inhibitors (LMWH and CMP)? These studies may provide new insight into the inhibition mechanism of tumor cell migration and lead to great improvements in infant formula for the gut health system.

## 2. Methods and Materials

### 2.1. Material Sources

The PSTD peptides were made according to previous studies [22,23]. 3′-SL and 6′-SL were purchased from BIOSYNTH Carbosynth (Suzhou, China). Their formulas are all C_23_H_38_NO_19_Na, and molecular weight is 655.53 g/mol.

### 2.2. Preparations of Circular Dichroism (CD) Samples and the Measurements and Recorded Methods of CD Spectra

The PSTD peptide (35 amino acids), SLs, CMP-Sia, and polySia (or PSA) were dissolved in 20 mM phosphate buffer (pH 6.7) with 25% tetrafluoroethylene (TFE), and their concentrations were 80 μM, 40 μM, 40 μM, and 4 μM, respectively. The measured and recording methods of CD spectra are described in previous articles [24,25,27,32].

### 2.3. Preparation of NMR Samples

All NMR samples were dissolved in 25% TFE (*v*/*v*), 10% D2O (*v*/*v*), and 65% (*v*/*v*) 20 mM phosphate buffer. pH 6.7. 2-dimethyl-2-silapentane-5-sulfonic acid (DSS) was used as the internal standard for measurements of the chemical shift. The concentrations of the PSTD in the absence and presence of the ligands were 2 mM, and the concentrations of 3′-SL and 6′-SL in the buffers were 0.5 mM, 1.0 mM, 2.0 mM, and 3.0 mM, respectively.

### 2.4. NMR Spectroscopic Measurements

NMR is a powerful biophysical tool for studying structures and functions of biomolecules, interactions of protein–DNA, and interactions of protein–ligands [33,34,35,36,37,38,39,40,41,42,43,44,45,46,47,48,49,50,51,52,53]. The experimental and measured methods of all NMR spectra were the same as described in previous articles [25,27].

A 2D HSQC spectrum can rapidly provide ^1^H and ^13^C, or ^1^H and ^15^N chemical shifts in each residue of a protein or peptide. The overlaid 2D HSQC spectra of the PSTD in the absence and presence of a ligand can accurately determine whether a ligand is bound to a specific residue in the PSTD [31]. In order to compare the binding affinity between different ligands and the PSTD, CSPs of all amino acids in the PSTD were calculated based on measured ^1^H and ^13^C, or ^1^H and ^15^N chemical shift values from the 2D HSQC spectra. The formula for CSP calculations is as follows [49,50,51,52,53]:

CSP = [(D2_NH_ + (D_N_/5)2)/2]1/2 (1)

where D_N_ and D_NH_ represent the changes in ^15^N and ^1^H chemical shifts, respectively, upon ligand binding [49,50,51,52,53].

Chemical shift perturbation (CSP) titration experiments are ideally suited for characterizing the binding interface of macromolecular complexes. ^1^H-^15^N-HSQC-based CSP studies have become the method of choice due to their simplicity, short time requirements, and not requiring high-level NMR expertise.

## 3. Results

### 3.1. CD Data

**Figure 1:**
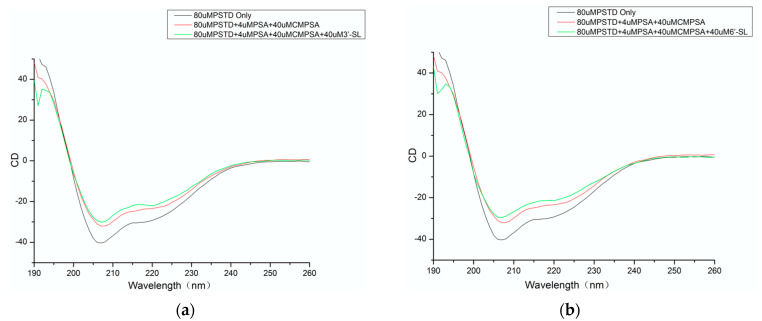
CD spectra of the PSTD alone (black), and in the presence of polySia/CMP-Sia (red), or in the presence of polySia/CMP-Sia/3′-SL (green) (**a**); CD spectra of the PSTD peptide in the absence of any ligand black and in the presence of polySia/CMP-Sia (red), or in the presence of polySia/CMP-Sia/6′-SL (green) (**b**).

As shown in Figure 1 and Table 1, 26.5% of the PSTD structure displayed α-helices before a ligand was added into the CD sample, and the α-helical content was changed to 18.1% in the presence of CMP-Sia and polySia. The helical contents were slightly decreased to 17.7% after 3′-SL was added to the mixture of the CMP-Sia and polySia (Figure 1a and Table 1). However, the helical contents decreased to 14.5% after 6′-SL was added into the mixture of the CMP-Sia and polySia (Figure 1b and Table 1). These results suggest that 3′-SL or 6′-SL might be bound to the H2 α-helical domain.

### 3.2. NMR Results

**Figure 2:**
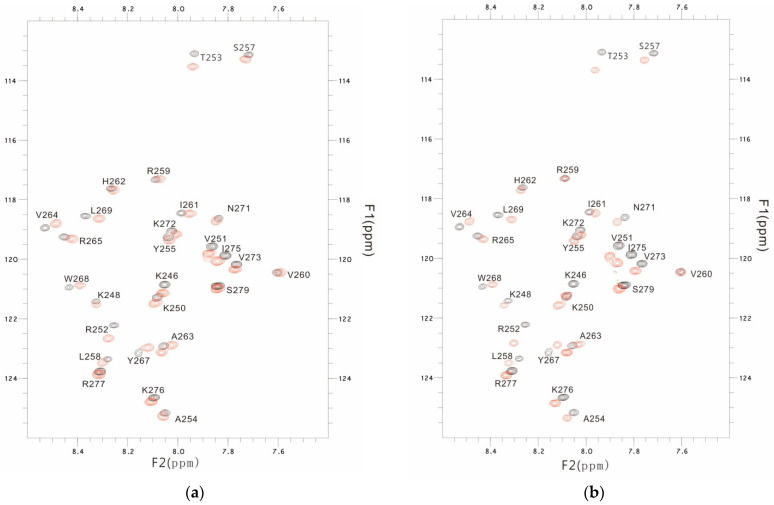
The overlaid ^1^H-^15^N HSQC spectra of the PSTD in the absence and presence of 0.5 mM 3′-SL (**a**), and 1 mM 3′-SL (**b**), respectively. The obvious changes in chemical shift are residues located in the N-terminus and C-terminal, comparing (**a**) with (**b**).

**Figure 3:**
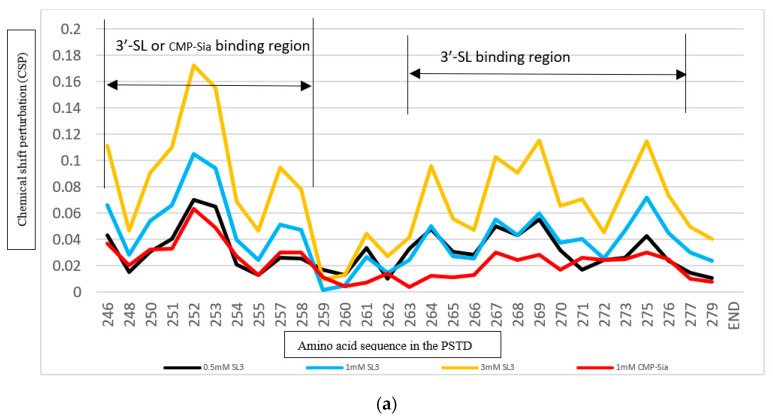

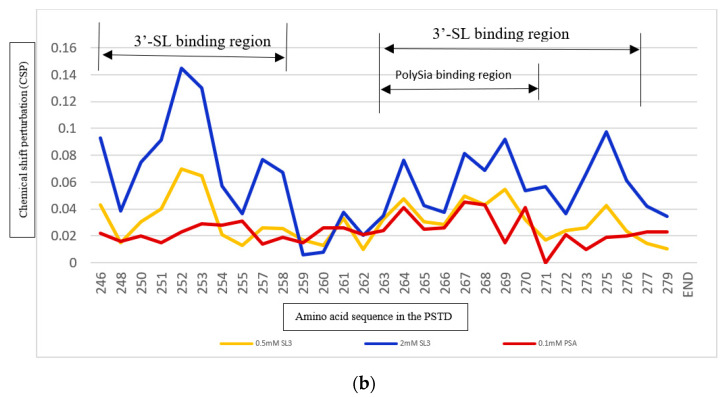
CSPs of the PSTD for the PSTD-(CMP-1 mM Sia), the PSTD-(0.5 mM 3′-SL), the PSTD-(1 mM 3′-SL), and the PSTD-(3 mM 3′-SL) interactions, respectively (**a**); the CSPs of the PSTD for the PSTD-(0.1 mM polySia or PSA), the PSTD-(0.5 mM 3′-SL) and the PSTD-(2 mM 3′-SL) interactions, respectively (**b**).

**Figure 4:**
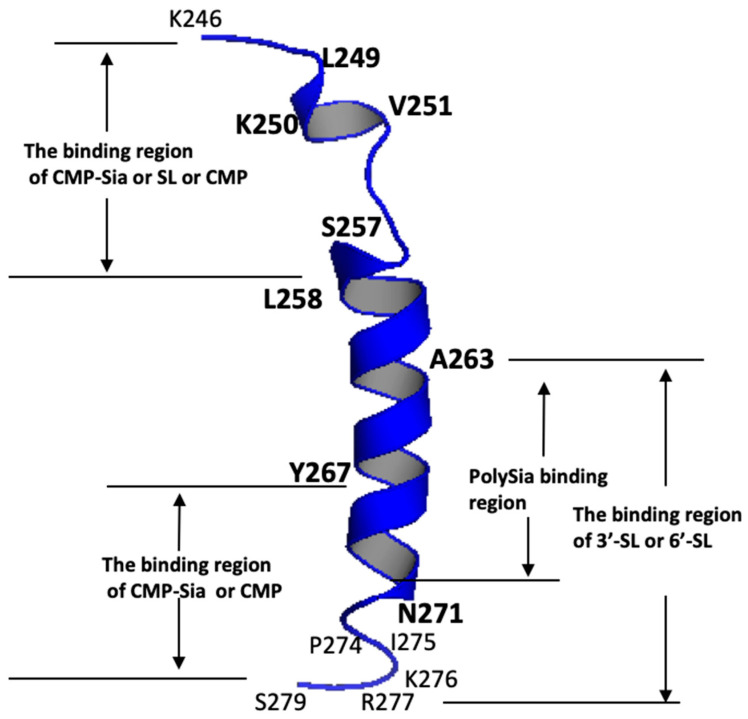
The binding ranges of the three ligands, CMP-Sia, 3′-SL and 6′-SL on the PSTD based on the results shown in Figure 2, Figure 3, Figure 5 and Figure 6, respectively.

The changes in the chemical shift in the PSTD were detected using the overlaid HSQC spectra in the absence or presence of 3′-SL (Figure 2), and chemical shift perturbations (CSPs) were further found in 17 residues (Figure 3), which cover the N-terminus of the PSTD, the short helix (L249–V251), the flexible region (R252–S257) between the short helix and the long helix, the C-terminus (V264–N271) of the long helix H2, as well as the C-terminal PSTD (V273–R277) (Figure 4).

As shown in Figure 3, the CSP values in the above regions are larger for the PSTD-(1 mM 3′-SL) interaction than for the PSTD-(0.5 mM 3′-SL) interaction but smaller than for the PSTD-(3 mM 3′-SL) interaction (Figure 3a). In addition, we also found that the CSP values for PSTD-(0.5 mM 3′-SL) interaction are almost equal to that for the interaction between the PSTD and CMP-Sia in the binding region of CMP-Sia (K246–L258) (Figure 3a), which covers the N-terminus of the PDTD, the flexible region between short helix H1 and long helix H2 (Figure 4). These findings suggest that PSTD-(CMP-Sia) binding could be inhibited when the 3′-SL concentration is more than 0.5 mM.

The CSP values of the PSTD between the PSTD-polySia interaction and the PSTD-(3′-SL) interaction can be compared using Figure 3b, in which the CSP values for the PSTD-(0.5 mM 3′-SL) are very close to that for the PSTD-polySia binding in a residue range from 263 to 271. In addition, polySia binding sites (A263–N271) are also covered by the binding region of 3′-SL binding (A263–R277) (Figure 3b). These results suggest that the interactions between the PSTD and CMP-Sia, and the PSTD and polySia, are inhibited when the 3′-SL concentration is at least 0.5 mM.

### 3.3. The PSTD-(6′-SL) Interaction

**Figure 5:**
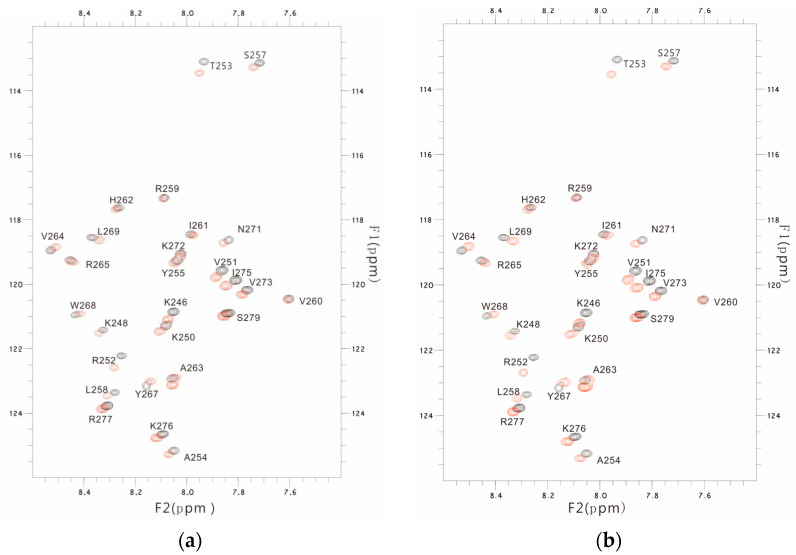
The overlaid ^1^H-^15^N HSQC spectra of the PSTD in the absence and presence of 1.0 mM 6′-SL (**a**), and 2.0 mM 6′-SL (**b**), respectively. The obvious changes in chemical shift are residues K246, R252, T253 in the N-terminus, comparing (**a**) and (**b**).

**Figure 6:**
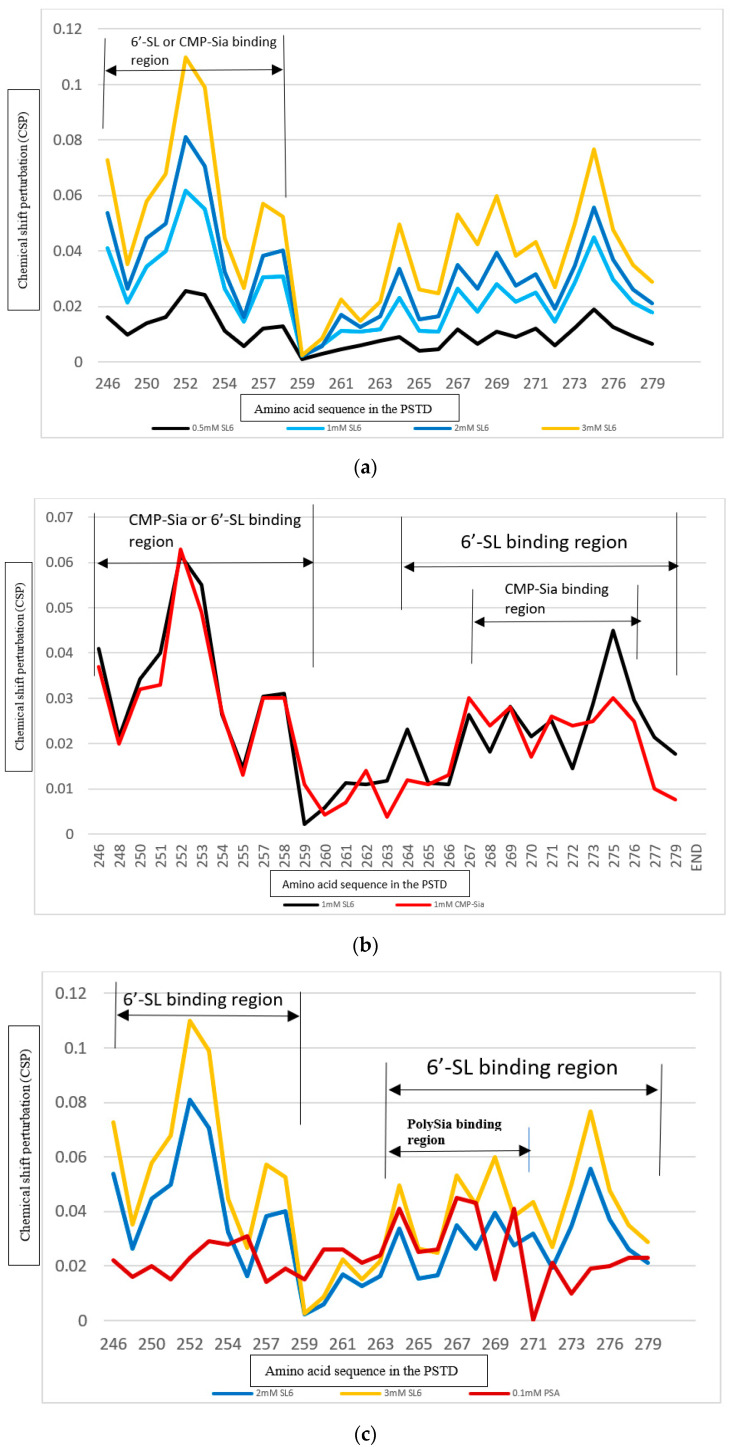
The chemical shift perturbations (CSPs) of the PSTD in the presence of 0.5, 1.0, 2.0, and 3.0 mM 6′-SL (**a**); the CSPs of the PSTD when interacting with 1 mM CMP-Sia, and 1.0 mM 6-SL, respectively (**b**); and the CSPs of the PSTD when interacting with 0.1 mM PSA, 2.0 mM and 3 mM 6′-SL, respectively (**c**).

In order to study the effects of 6′-SL on polysialylation, 2D overlaid ^1^H-^15^N HSQC experiments of the PSTD in the absence and presence of 6′-SL (6′-SL’s concentration 0.5 mM, 1 mM, and 2 mM, respectively) were carried out to determine the effects of 6′-SL on polysialylation. The overlaid HSQC spectra of the PSTD in the presence of 6′-SL (1.0 mM) and 6′-SL (2.0 mM) are shown in Figure 5. Similar to Figure 2, more changes in chemical shift were observed for most amino acids after the 6′-SL concentration was increased to 2.0 mM (Figure 5b) from 1.0 mM (Figure 5a), and the CSPs of the amino acids in the PSTD-(CMP-Sia) binding region were also increased with 6′-SL concentrations from 0.5 mM to 3 mM (Figure 6a).

An unexpected finding is that the CSPs of the interaction between the PSTD and the PSTD-(CMP-Sia) are almost the same as for the PSTD-(1.0 mM 6′-SL) interaction in the two residue ranges, from 246 to 258 and 265 to 273 (Figure 6b). However, the CSP values of the PSTD for the PSTD-polySia interaction are very close to the median values of the CSPs between PSTD-(3 mM 6′-SL) binding and PSTD-(2 mM 6′-SL) binding (Figure 6c). Thus, the above results suggest that both PSTD-(CMP-Sia) and PSTD-polySia binding should be inhibited when the 6′-SL concentrations are 1 and 3 mM, respectively.

### 3.4. Comparison of the Inhibition of the SLs and Heparin (LMWH) on Polysialylation

#### 3.4.1. Comparison of the Inhibition of the SLs and Heparin (LMWH) on the PSTD-CMP-Sia Interaction

Recent NMR studies indicate that LMWH can inhibit NCAM polysialylation, and the binding sites of LMWH on the PSTD were located on the N-terminus, the long helix H2, and the C-terminus of the PSTD [26]. 

It is known that NMR chemical shift perturbation (CSP) data, routinely obtained using 2D ^1^H-^15^N HSQC spectra, follow changes in the chemical shifts of a protein or peptide when a ligand is added, and these are used to determine the location of the binding site, the affinity of the ligand, and/or possibly the structure of the complex [54]. Therefore, the binding affinity between a protein (or peptide) and two different ligands can be determined through comparison of CSPs in the same binding region of these two ligands on the protein or peptide [54]. More recent NMR studies verified that the CSPs of the PSTD for the interaction between the PSTD and LMWH are larger than that for the interactions between the PSTD and CMP-Sia, and the PSTD and polySia. These results further confirmed that LMWH is an effective inhibitor for inhibitions of the PSTD-(CMP-Sia) and PSTD-polySia [32]. 

**Figure 7:**
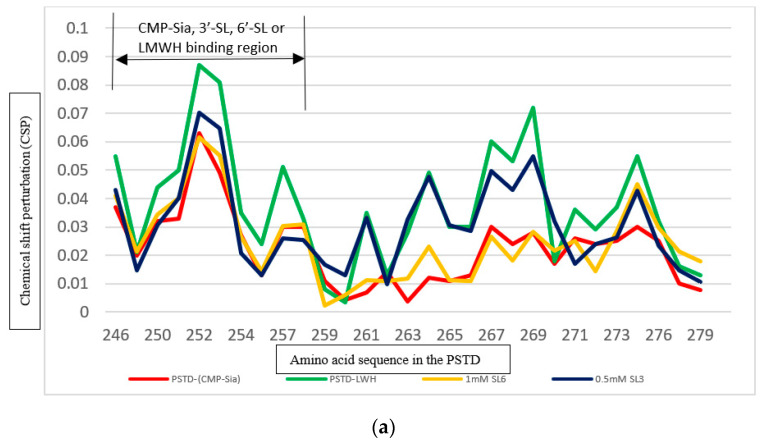

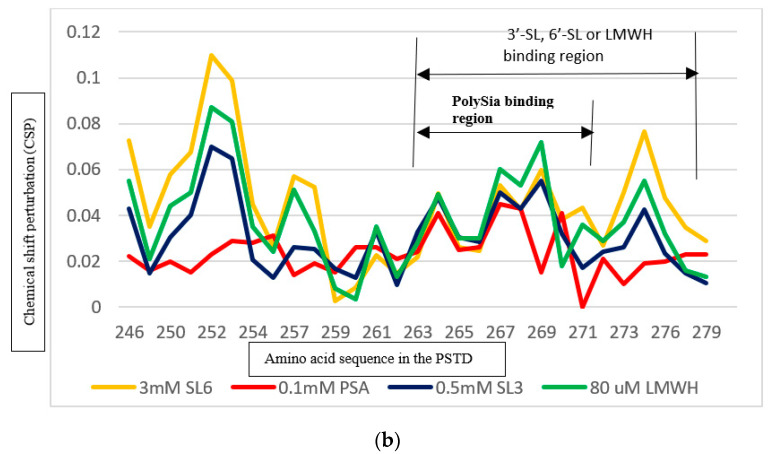
Comparison of the CSPs in the PSTD when PSTD interacted with different ligands. The CSPs of the PSTD for the PSTD-0.5 mM 3′-SL, the PSTD-1 mM 6′-SL, the PSTD-1 mM (CMP-Sia), and the PSTD-80 μM LMWH interactions (**a**); CSPs of the PSTD for the PSTD-0.5 mM 3′-SL, the PSTD-3 mM 6′-SL, the PSTD-80 μM LMWH, and the PSTD-0.1 mM polySia interactions (**b**).

The LMWH binding region is almost the same as that of 3′-SL and 6′-SL in the PSTD (Figure 7). The CSPs of the PSTD for the PSTD-LMWH (80 μM) and PSTD-SL (0.5 mM 3′-SL or 1mM 6′-SL) interactions are all larger than that for the interaction between the PSTD and the PDTD and CMP-Sia (Figure 7a). These results suggest that 0.5 mM 3′-SL or 1mM 6′-SL or 80 μM LMWH can inhibit PSTD-(CMP-Sia) binding. Comparing the STD-LMWH (80 μM) interaction with the PSTD-SLs interactions (Figure 7a), the CSPs of the former are larger than that of the latter (Figure 7a), indicating stronger inhibition of LMWH than SLs.

#### 3.4.2. Comparison of the Inhibition of the SLs and Heparin (LMWH) on the PSTD-polySia Interaction

It is known that 0.1 mM polySia could be bound to four residues, R265, Y267, W268, and L269, in the H2 helix of the PSTD [23,24,25]. In this study, we found that the CSPs for the PSTD-LMWH (80 μM), PSTD-(0.5 mM 3′-SL), and PSTD-(3 mM 6′-SL) interactions are larger than that for that for the interaction between polySia and the PSTD in the R265-N271 region, particularly from residues V264 to L269 (Figure 7b), suggesting the PSTD-polySia interaction could be inhibited by LMWH or 3′-SL or 6′-SL. Specifically, the CSPs for PSTD-(0.5 mM 3′-SL) and the PSTD-(3 mM 6′-SL) interactions are less than that of the PSTD-LMWH (80 μM) in the common binding range A263–N271 between these three ligands and the PSTD.

### 3.5. Comparison of the Inhibition of the SLs and CMP on Polysialylation

#### 3.5.1. Comparison of the Inhibition of the 3′-SL and CMP on the PSTD-(CMP-Sia) Interaction

Previous in vitro experiments showed that the polysialylation (trimer) of α-2,8-linked sialic acid (triSia) could be inhibited by CMP [55,56]. Recent NMR studies confirmed that CMP is able to inhibit PSTD-(CMP-Sia) interaction but not PSTD-polySia interaction [27].

The residue ranges K246–L258 and Y267–R277 are two binding regions of CMP-Sia in the PSTD (Figure 4). The first binding region (K246–L258) is also the binding domain of CMP or SLs (Figure 4), and the second binding region (Y267–R277) shares the same binding region with CMP. However, the Y267–R277 region is covered by the second binding region of SLs (A263–R277) (Figure 4).

**Figure 8:**
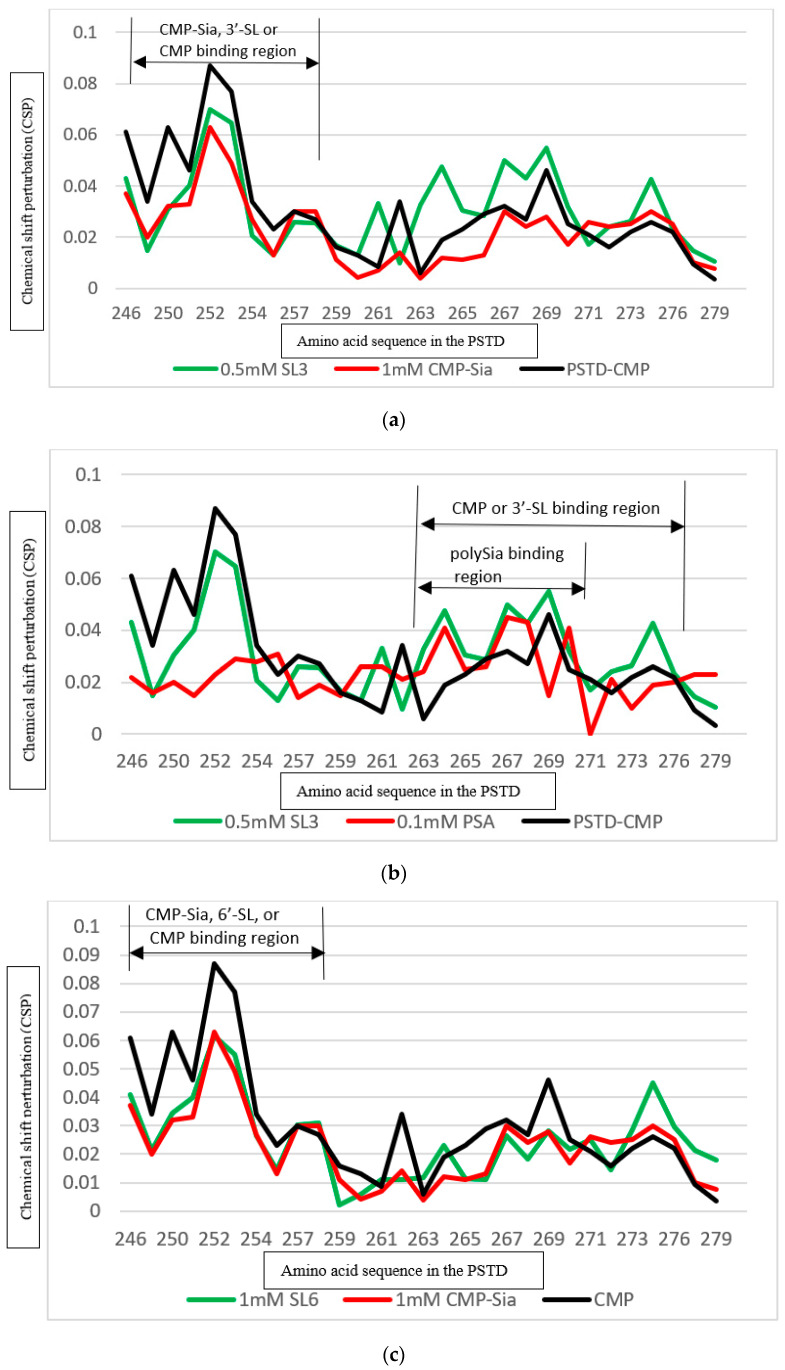

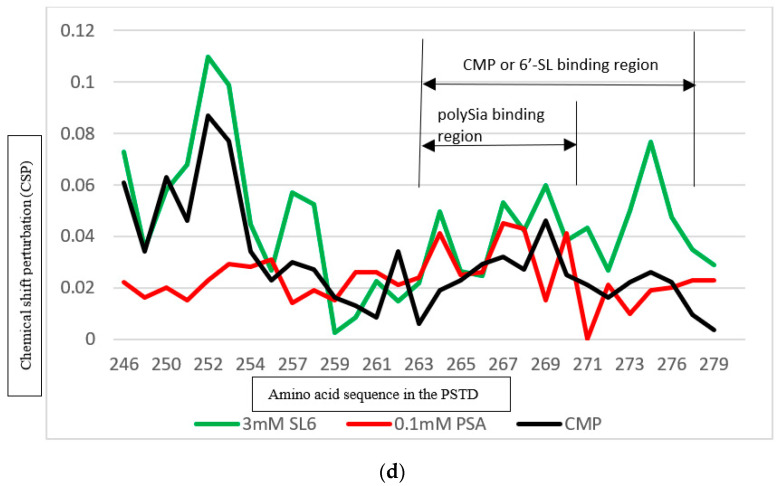
Comparison of the inhibitions of the SL CMP for the interaction between the PSTD and CMP-Sia, and the interaction between the PSTD and polySia. The CSPs of the PSTD for the PSTD-(0.5 mM 3′-SL), the PSTD-1 mM CMP, and the PSTD-(1 mM CMP-Sia) interactions (**a**); CSPs of the PSTD for the PSTD-(0.5 mM 3′-SL), the PSTD-1 mM CMP, and the PSTD-(0.1 mM polySia) interactions (**b**); CSPs of the PSTD for the PSTD-(1 mM 6′-SL), the PSTD-1 mM CMP, and the PSTD-(1 mM CMP-Sia) interactions (**c**); CSPs of the PSTD for the PSTD-(3 mM 3′-SL), the PSTD-1 mM CMP, and the PSTD-(0.1 mM polySia) interactions (**d**).

As shown in Figure 8a, the CSPs for PSTD-(0.5 mM 3′-SL) interaction are larger than that for the interaction between the PSTD and 1mM CMP-Sia in the main PSTD-CMP binding interaction region of CMP-Sia in the PSTD (K246–L258) (Figure 8a). This suggests that 0.5 mM 3′-SL is a more effective inhibitor in inhibiting the binding of CMP-Sia to the PSTD.

It has been proposed that the binding sites of polySia are at A263–N271 of the long helix of the PSTD (Figure 4) [23,24,25,27], and the CSPs for the interaction between the CMP and the PSTD are less than that for the PSTD and polySia (Figure 8b). This result suggests that CMP is not able to inhibit the interaction between the PSTD and polySia.

However, the chemical shift perturbation values are larger for the interaction be-tween the PSTD and 0.5 mM 3′-SL than for the interaction between the PSTD and polySia (Figure 8b). This comparison further suggested that 0.5 mM 3′-SL is also an ideal inhibitor in inhibiting PSTD-polySia interaction.

#### 3.5.2. Comparison of the Inhibition of the 6′-SL and CMP on Polysialylation

It has been proposed that the PSTD-(CMP-Sia) interaction could be inhibited when the 6′-SL concentration is above 1 mM (Figure 6b). However, the CSP values of all residues, except L258 in the CMP binding region of the PSTD, are lower for the interaction between the PSTD and 1 mM 6′-SL than that for the interaction between the PSTD and 1 mM CMP (Figure 8c).

In order to make the CSPs for the PSTD-(6′-SL) larger than for the PSTD- 1 mM CMP interaction, the concentration should be about 3 mM (Figure 8d). 

As shown in Figure 8c, the CSPs of the PSTD-(CMP-Sia) and the PSTD-6′-SL(1 mM) are very close to each other in the binding region from residue 246 to 258. This suggests that more than a 1 mM concentration of 6′-SL will inhibit the PSTD-(CMP-Sia) interaction. 

Previous studies have shown that the interaction between the PSTD and polySia cannot be inhibited by CMP [27]. Compared with CMP, the CSPs for the PSTD-(3 mM 6′-SL) interaction are larger than that for the PSTD-polySia interactions (Figure 8d). Thus, it has been suggested that the PSTD-polySia interactions could be inhibited by 3 mM 6′-SL.

## 4. Discussion

The PSTD-(CMP-Sia) and the PSTD-polySia interactions have been verified in previous NMR studies [24,25], and the largest chemical shift perturbations (CSPs) for the PSTD-(CMP-Sia) interaction were mainly distributed in the N-terminal region that included a short H1 helix (L249–V251), and the flexible region between the short helix and the N-terminus of the long helix H2 (K246–L258). In addition, the largest CSP for the interaction between the PSTD and polySia is mainly distributed in the long H2 helical domain of the PSTD, such as at residue positions A263, R265, Y267, and L269 [24,25], which are also consistent with the prediction results using Chou’s Wenxiang diagrams [57,58,59,60,61,62,63]. In this study, the helical contents of the PSTD in the absence of any ligands were 26% and were decreased to 18.4% in the presence of the mixture of CMP-Sia and polySia. It is possible that the major contribution of the α-helical content reduction is due to the formation of polySia chain binding to the long helix H2, inducing the partial unwinding of the helix. Because CMP-Sia cannot be bound to the long helix H2, the helical contents of the PSTD were decreased to 17.7% or 14.5%, after 0.5 mM 3′-SL or 3 mM 6′-SL was added to the mixture of the CMP-Sia and polySia. Similar to polySia, these results suggest that SLs could also be bound to the long helix H2 of the PSTD, playing an important role in unwinding the long helix H2.

The analysis results of the above CD spectra are further supported by our NMR experiments. As shown in Figure 2, Figure 3, Figure 4 and Figure 5, the SL binding regions for the PSTD-(3′-SL) interaction or the PSTD-(6′-SL) interaction are in two ranges, K246–L258 and A263–R277. The former (K246–L258) is also the main binding region of CMP-Sia, and the latter (A263–R277) covers the binding region (A263–N271) of the PSTD-polySia interaction.

In this study, the CSPs are not only larger for the interaction between the PSTD and 3′-SL than that for the interaction between PSTD and (CMP-Sia) (Figure 3a) but also larger than that for the PSTD-polySia binding region (A263–N271) (Figure 3b) when the 3′-SL concentration is higher than 0.5 mM; thus, it was suggested that both binding between the PSTD and (CMP-Sia) and binding between the PSTD and polySia can be simultaneously blocked using 0.5 mM 3′-SL (Figure 3 and Table 2). Here, an unexpected finding is that the concentration of 3′-SL in human milk during lactation is 220 mg/L [9], which is very close to 0.5 mM. This consistency also reflects that the concentrations of 3′-SL should be stable during the lactation period [9,64]. The 6′-SL concentration should be higher than 1 mM in order to make the CSPs for the interaction between the PSTD and 6′-SL larger than that for the interaction between the PSTD and CMP-Sia in the common binding region of 6′-SL and (CMP-Sia) on the PSTD (K246–L258) (Figure 6b), and it takes around 3 mM 6′-SL to make the CSPs for the PSTD-(6′-SL) interaction larger than that for the PSTD-polySia interaction in their common binding region (A263–N271) (Figure 6c and Table 2).

Although 3′-SL and 6′-SL have similar biological functions in inhibiting the growth of pathogens [9], promoting the growth of beneficial intestinal bacteria [10] and boosting neural function and cognitive development [6], there is an obvious difference between these two SLs in the concentration levels for the supplement of the infant formula and the inhibition of polysialylation. The above results show that the same low 3′-SL concentration (0.5 mM) could be used to inhibit the interaction between the PSTD and (CMP-Sia) and the interaction between the PSTD and polySia. However, concentrations of 6′-SL are more than that of 3′-SL to inhibit these two interactions. This difference suggests that the consumption of 6′-SL might be higher than that of 3′-SL and the 6′-SL concentration level’s instability for gut and neural system developments.

In addition, a recent study also suggested that adjustments in cognitive development in maternal milk need a upregulation of neuronal patterning [65]. Thus, this analysis may explain why the 6′-SL concentration in human milk is reduced to 250 mg/L at 2 months from 500 mg/L at 1 month (Table 2) [9] and why the supplement of infant formula for 6′-SL should be more than that for 3′-SL.

As shown in Figure 7a, the chemical shift perturbations (CSPs) for the interaction between the PSTD and CMP-Sia are less than that for the interaction between the PSTD and 80 μM LMWH (low-molecular-weight heparin), suggesting that the binding of the PSTD and CMP-Sia could be inhibited using 80 μM LMWH, in addition to using 0.5 mM 3′-SL or 1 mM 6′-SL. In the other hand, the CSPs of the PSTD-polySia interaction are less than that of the PSTD-(80 μM LMWH), and, thus, it is suggested that PSTD-polySia binding could be inhibited by 80 μM LMWH, in addition to 0.5 mM 3′-SL or 3 mM 6′-SL (Figure 7b). Among the interactions of the PSTD-(80 μM LMWH), the PSTD-(0.5 mM 3′-SL), and the PSTD-(3 mM 6′-SL), the largest CSPs were displayed in the PSTD-(80 μM LMWH) interaction in both the CMP-Sia binding region and polySia binding region. Although LMWH seems to be a more powerful inhibitor compared with the SLs, it should be used with care in clinical research and use. Coagulation, development, the stabilization of fibrin clots, and severe bleeding are often related to high-dose heparin in the clotting cascade [66].

It has been proposed that NCAM polysialylation inhibition could be achieved through inhibiting the binding between the CMP-Sia and the PSTD or the interaction between polySia and the PSTD [23,24]. In actual operation, it is difficult to determine when polySia has been formed in the system. Therefore, the control of the inhibition for PSTD-(CMP-Sia) binding should be more easy and efficient than that for PSTD-polySia binding in the early stages of polysialic acid formation. Our recent NMR studies showed that the inhibition of polysialylation could be partially inhibited by CMP [27] because CMP can only inhibit PSTD-(CMP-Sia) interaction but not PSTD-polySia interaction. Therefore, CMP has been proposed to be a competitive inhibitor of polySTs and might be useful for the regulation of the life process.

Comparing SLs with CMP, not only the interaction between the PSTD and (CMP-Sia) but the interaction between the PSTD and polySia can be inhibited by SLs (3′-SL or 6′-SL). Moreover, SLs have been verified to be the main components of novel food (NF), which could be added to infant formula and food supplements (FSs) without safety concerns, according to the right conditions for use [67,68,69]. These analyses displayed the bifunctional effects of SLs, which may benefit from a stronger focus on CAMM (Computer-Assisted Molecular Modelling) methods [70,71,72] for novel drug design.

In the Introduction, we mentioned that it is possible that SL, Sia, CMP-Sia, polySia, and polyST coexist when polySia is produced from CMP-Sia. It is possible that polyST may not only individually interact with CMP-Sia or polySia but also be directly in contact with the mixture of CMP-Sia and polySia. 

Recent NMR studies showed that CMP-Sia is mainly bound in a residual range from K246 to L258 in the PSTD, but polySia is bound to the H2 helix of the PSTD and the obvious changes in chemical shifts were at four residues, V264, Y267, W268, and L269 [23,25], when the PSTD, CMP-Sia, and polySia coexist in the solution system. These results supported the previous hypothesis [25,27]. 

Our NMR results also displayed that the CSPs of the residues in the PSTD are larger for the PSTD-CMP interaction than that for the mixture of the PSTD, polySia, and CMP-Sia in a residual range from K246 to L258 (the binding region of CMP-Sia in the PSTD) but smaller than that for the interaction between the PSTD and polySia in a residual range from V260 to N271 (the binding range of polySia in the PSTD) [27]. These results verified that CMP can only be bound to the binding region of CMP-Sia in the PSTD when CMP-Sia, polySia, CMP, and the PSTD were mixed in the solution.

The above results also display that the CSPs of the PSTD are larger for the mixture of the PSTD, polySia, and CMP-Sia than that for the mixture of the PSTD and polySia in a residual range from I263 to T270 (Figure 3b), and the decreases in peak intensity were also found at nine residues, S257, L258, R259, I261, R265, Y267, W268, L269, and N271, when the PSTD, CMP-Sia, and polySia were mixed together. In contrast, the decreases in peak intensity were displayed at 20 residues for the mixture of the PSTD and polySia [25,27]. This obvious differences in the decrease in peak intensity suggest that CMP-Sia may decrease the gathering level of polySia in the PSTD. 

In future studies, CSPs of the PSTD for PSTD-SL interaction and CSPs of the PSTD for the PSTD-polySia/CMP-Sia mixture interaction will be further compared and studied in order to determine the effects of other inhibitors (SLs or LMWH) when they coexist in a mixture of CMP-Sia and polySia.

## 5. Conclusions

The selection of 3′-SL suitable for infant formula during lactation is mostly based on the following two factors:

First, it is known that the major SL in humans is 6′-SL, and its concentration level is rapidly declined during lactation. In contrast, 3′-SL is predominant in infant formula, and its concentration remains relatively stable [7].

Second, the biological functions of 3′-SL and 6′-SL are similar in protecting against pathogens [8], promoting the growth of beneficial bacteria in the gut, and enhancing neural function and cognitive development [5].

In this study, our NMR results, summarized in Table 2 and Table 3, further verified that the 3′-SL concentration is more stable compared to 6′-SL, and the interactions between the PSTD and CMP-Sia, and between the PSTD and polySia, could be inhibited using 0.5 mM 3′SL. 

However, considering sialylation in milk, but not in the cells (digestion, transfer, etc., losses), this 0.5 mM theoretical value may need to be appropriately increased to make this supplement suitable for infant formula and inhibiting NCAM polysialylation overexpression.

Although LMWH is a powerful inhibitor of polysialylation compared with CMP and SLs according to the data in Table 3, its high-dose use often induces serious problems in terms of the clotting cascade and neural system development. SLs are more powerful inhibitors than CMP and do not raise any safety concerns, whether as a novel food or inhibitor of polysialylation in a larger concentration range. The bifunctional effects of SLs maintain an optimum balance between gut system health and neural system developments. Such a balance could avoid the formation of the excessed polySia chains and, thus, may block tumor metastasis in cancer patients [73,74,75,76,77,78,79,80,81,82,83,84,85]. As Toman’s Law states, “Enough of Anything will Inhibit Anything” [86].

## Figures and Tables

**Table 1:** The helix content of the PSTD alone or after a ligand was mixed with the PSTD.

The helix content of the PSTD alone (%): 26.5	The helix content of the PSTD after CMP-Sia and polySia were added (%): 18.4
26.5	The helix content of the PSTD after CMP-Sia, polySia and 3′-SL were added (%): 17.7
26.5	The helix content of the PSTD after CMP-Sia, polySia and 3′-SL were added (%): 14.5

**Table 2:** The differences of 3’-SL and 6’-SL in the required concentrations for binding to the PSTD based on the NMR experiments. The measured maximum concentrations of SLs during 1st month of the lactation, and the SL’s concentrations when the chemical shift perturbations (CSPs) for the PSTD-SL (3′-SL or 6′-SL) binding are close to that for the PSTD-(CMP-Sia) binding and the PSTD-polySia binding, respectively.

Sialylactose (SL) interacted with the PSTD	SL’s concentration when CSP values of PSTD-SL binding are close to that of PSTD-(CMP-Sia) binding in same amino acid range of the PSTD (mM)	SL’s concentration when CSP values of PSTD-SL binding are close to that of PSTD-polySia binding in same amino acid range of the PSTD (mM)	The maximum concentration of the SL in human milk during 1st month of lactation (mM) [9]
3′-SL	0.5	0.5	Ca. 0.5
6′-SL	1.0	Ca. 3.0	Ca. 1.0

**Table 3:** Summary of the binding regions of the ligands CMP-Sia, polySia, LMWH, CMP, 3′-SL (0.5 mM) and 6′-SL (1 mM) on the PSTD, the largest CSPs of each binding region, and CMP-Sia and polySia binding regions are covered by the binding regions of other ligands on the PSTD. CSPs were obtained based on the 2D ^1^H-^15^N HSQC experiments.

Ligands Binding to the PSTD	The Main Binding Regions of the Ligands in the PSTD	The Maximum CSPs of Each Binding Region
CMP-Sia	K246–S257 V267–K276	0.070 0.026
polySia	A263–N271	0.049
Heparin LMWH	K246–S257 A263–R277	0.087 0.072
CMP	K246–S257 A263–R277	0.087 0.046
3′-SL (0.5 mM)	K246–S257 A263–R277	0.070 0.043
6′-SL (1 mM)	K246–S257 A263–R277	0.070 0.026

## Data Availability

The data presented in this study are available on request from the corresponding author.

## Abbreviations

polySia	polysialic acid
Sia	mono-sialic acid
CMP-Sia	cytidine monophosphate-sialic acid
NCAMs	neural cell adhesion molecules
polySTs	polysialyltransferases (ST8Sia II (STX) & ST8Sia IV (PST)
PSTD	polysialyltransferase domain
SL	sialyllactose
SMO	sialylated milk oligosaccharide
HMO	human milk oligosaccharide
CSP	chemical shift perturbation
